# Early Childhood Reading in Rural China and Obstacles to Caregiver Investment in Young Children: A Mixed-Methods Analysis

**DOI:** 10.3390/ijerph18041457

**Published:** 2021-02-04

**Authors:** Rui Li, Nathan Rose, Yi Ming Zheng, Yunwei Chen, Sean Sylvia, Henry Wilson-Smith, Alexis Medina, Sarah-Eve Dill, Scott Rozelle

**Affiliations:** 1 Rural Education Action Program (REAP), Freeman Spogli Institute for International Studies, Stanford University, Stanford, CA 94305, USA; lirui.uw@gmail.com (R.L.); natescience@yahoo.com (N.R.); yiming0129@yahoo.ca (Y.M.Z.); henry.wilsonsmith@gmail.com (H.W.-S.); amedina5@stanford.edu (A.M.); rozelle@stanford.edu (S.R.); 2 Department of Health Policy and Management, Gillings School of Global Public Health, University of North Carolina at Chapel Hill, Chapel Hill, NC 27599, USA; ywchen@live.unc.edu (Y.C.); sean.sylvia@unc.edu (S.S.)

**Keywords:** early childhood development, stimulating parenting practices, effective early childhood reading practices, rural China, mixed methodology

## Abstract

Studies have shown that nearly half of rural toddlers in China have cognitive delays due to an absence of stimulating parenting practices, such as early childhood reading, during the critical first three years of life. However, few studies have examined the reasons behind these low levels of stimulating parenting, and no studies have sought to identify the factors that limit caregivers from providing effective early childhood reading practices (EECRP). This mixed-methods study investigates the perceptions, prevalence, and correlates of EECRP in rural China, as well as associations with child cognitive development. We use quantitative survey results from 1748 caregiver–child dyads across 100 rural villages/townships in northwestern China and field observation and interview data with 60 caregivers from these same sites. The quantitative results show significantly low rates of EECRP despite positive perceptions of early reading and positive associations between EECRP and cognitive development. The qualitative results suggest that low rates of EECRP in rural China are not due to the inability to access books, financial or time constraints, or the absence of aspirations. Rather, the low rate of book ownership and absence of reading to young children is driven by the insufficient and inaccurate knowledge of EECRP among caregivers, which leads to their delayed, misinformed reading decisions with their young children, ultimately contributing to developmental delays.

## 1. Introduction

Neurological research has indicated that most brain development occurs within the first three years of a child’s life [1], and strong cognitive development during this critical period is a key predictor of education, employment, and earning power in later life [2]. Moreover, early childhood development has important impacts on children’s future behavior, physical and mental health, and non-cognitive abilities, including motivation and self-confidence [3]. In the United States, huge disparities in children’s cognitive development exists between lower- and higher-income families, with resulting cognition gaps developing as early as 18 months [1]. These early cognition gaps have been shown to have lifelong, potentially irreversible effects [4].

Previous studies have shown that during this critical period (the first three years), it is important that caregivers provide their babies with stimulating activities to support healthy brain development [1]. In particular, scholars have demonstrated that interactive reading during this critical period supports early childhood cognitive development and can lower levels of cognitive delay in children [5,6,7,8,9,10,11,12,13]. Compared to more standard reading practices, whereby parents simply read the text aloud or describe pictures or objects for their children, an interactive reading method is collaborative in nature. That is, caregivers actively engage their children to interact with the reading materials by asking children open-ended questions and suggesting potential responses to those questions. This open-ended, collaborative style has been shown to improve early childhood cognitive development [14,15,16].

Effective reading practices do not only involve an interactive reading style; age-appropriate reading materials are also crucial for effective stimulation. Due to the high pace of cognitive development before 3 years of age, the most effective types of reading materials vary for different age levels. For example, studies have shown that, during the first 6 to 12 months, object-labeling (using books that simply identify familiar objects and simple colors) is appropriate in facilitating children’s development [17,18]. Reading that same object-labeling book to a 2-year-old child, however, can reduce that child’s opportunity to become engaged in more complex learning and social interactions. At age 10 months to 2 years, concept books (books that explain and explore a specific topic, such as cars or animals) are more helpful for stimulating cognitive development [17]. Interactive, lift-the-flap, and rhythm/rhyme books can also help children develop motor skills and introduce sounds and syllables during this period [17,18]. Around 13 months, theme books (books that focus on sequentially related events and/or illustrations) can be introduced, and after around 19 months, plot-based stories can be introduced to teach more complex and extended language structures.

In this paper, we define effective early childhood reading practices (EECRP) as practices in which caregivers use age-appropriate reading materials and employ an interactive reading style for their children during the critical first 3 years. The knowledge of EECRP does not come naturally, however, and requires caregivers to receive specific training from an early childhood development center or through in-home visits for training on early childhood care [16]. Additionally, there is evidence that reading skills are poor overall in rural China, which suggests that some parents may not have the necessary reading skills to engage in EECRP with their children. In a recent large-scale study of reading skills conducted in 2015 among rural elementary school students in three provinces of China using an international reading assessment (the Progress in International Reading Literacy Study), the authors found that students in rural China rank last of all countries assessed in terms of reading skills [19]. While less is known about adult reading skills in rural China, this research suggests that the reading skills of rural caregivers (who attended school in decades prior to the study when rural schooling was of even lower quality) are similarly poor.

Despite the demonstrated importance of EECRP [16], however, only a limited number of studies have empirically examined the trends of EECRP in rural China. To the best of our knowledge, there are no studies that use primary source data to study the potential factors that prevent caregivers from providing EECRP for their children at a younger age. There are also no studies that measure the extent of the association between EECRP and cognitive outcomes.

The absence of studies on EECRP and cognition in rural areas of China is certainly not due to the lack of the need to study this serious problem. Empirical research has shown that there is a huge gap in China between urban and rural children in terms of levels of cognitive development. Studies have shown that nearly half of rural toddlers have cognitive delays, and more than half of rural toddlers exhibit language delays, due to an absence of interactive parenting practices such as interactive reading [9,13,20,21]. These early developmental delays have significant implications for the future labor force and productivity, as these delays mean that rural residents might not have equal access to increasing economic and educational opportunities, which can widen the existing gaps between rural and urban residents [13,22,23]. However, few studies have explored in depth the factors underlying the observed low levels of stimulating parenting among China’s rural caregivers. Moreover, to our knowledge, no study has examined the factors that may limit caregivers in rural China from engaging in EECRP (and thus cause developmental delays among rural children). Consequently, there is an urgent need to understand reading habits and EECRP in rural China.

This study seeks to investigate and map EECRP in rural China by employing a mixed-methods approach to fulfill two main objectives. First, we aim to study the general prevalence, perceptions, and correlates of EECRP with children’s levels of cognitive development in rural China through a quantitative survey and empirical analysis of the data. Second, through qualitative interviews and field observations, we examine five hypothesized factors (described in the Qualitative Research section) to gain a detailed understanding of the potential constraints of EECRP in rural China. Through a combination of quantitative survey data and qualitative interview and observation data, we aim to produce a high-resolution picture of which caregivers and households in rural China read to their children, how they read, and why caregivers do or do not engage in EECRP.

## 2. Materials and Methods

### 2.1. Quantitative Research

#### 2.1.1. Sample Area and Selection

The data used in the quantitative portion of this study were collected from a set of counties in the Qinling mountain region of Northwest China. The province that we worked in is relatively rural, with about half of residents as living in rural areas in 2014 [24]. In this way, the sample region is representative of China as a whole, where about 43% of people live in rural areas [25]. The per capita income of rural residents in the sample province is around 9000 RMB (USD1300), about 3000 RMB (approximately USD400) less than the national median per capita income for rural areas (12,363 RMB, or USD1759) [26]. The sample province is also almost 100% ethnically Han (>99%), the ethnicity in China that accounts for 92% of the nation’s overall population [27]. Overall, the sample province is heavily rural, relatively poor, and almost fully Han, which is fairly representative of large parts of rural China. This study received ethical approval from the Stanford University Institutional Review Board (IRB Protocol 25743).

A four-step protocol was used to select sample households for this study. First, in the Qinling mountainous region of China, we selected all 22 nationally designated poverty counties. Second, in each county, the research team obtained a list of all townships. From this list of townships, those that either housed the county seat or had no villages with more than 800 people were excluded. Once the exclusion criteria were applied, 100 of the remaining townships were randomly selected for inclusion in this study. After the sample townships were chosen, one village per township was then randomly selected. In total, 100 villages were included in our study. Finally, within each sample village, all children within our target age range (6–24 months) and their caregivers were surveyed. The final sample consisted of 1748 caregiver–child dyads (1748 children plus 1748 caregivers) across 100 villages/townships in 22 counties.

#### 2.1.2. Data Collection

The survey for this study was conducted in August 2016. In each participating household, the primary caregiver was self-identified as the individual most responsible for the child’s care (which, more than 95% of the time, was either the child’s mother or paternal grandmother) and administered a survey by trained enumerators that collected information on demographic characteristics, book ownership rates, and EECRP from all sample households. In addition, enumerators asked caregivers about their knowledge of early childhood reading and perceived importance of early childhood reading.

The research team used three measures to track book ownership and reading practices among the sample. To measure book ownership, surveyors asked respondents how many books for children the household had. Reading practices were determined through two relevant questions: First, surveyors asked whether caregivers had read a book to their child in the last three days; second, as an alternative to reading a book, surveyors asked whether caregivers told a story to their child in the last three days. These measures were taken from the Family Care Indicators (FCI) questionnaire, a measure developed by the United Nations Children’s Fund (UNICEF) to assess the home environment of young children in developing countries [28]. Multiple studies have used these measures questions internationally and in China [29,30]. These questions not only addressed book availability, but also reading practices among the sample caregivers.

In addition to reading practices, the surveyors asked respondents about their perceptions of the importance of and knowledge about early childhood reading. For both measures, a 7-point Likert scale was used to measure responses. To measure perceptions of the importance of early childhood reading, surveyors asked caregivers to rank how important they believed that reading to their child was (1 = totally unimportant, 7 = extremely important). For ease of analysis, these responses were placed into two groups: relatively unimportant (1–3) and relatively important (5–7). Similarly, caregivers were asked how much they knew about EECRP (1 = no knowledge, 7 = absolutely confident of their knowledge). As with EECRP values, these responses were grouped into relative categories: relatively unsure (1–3) and relatively sure (5–7).

Child cognition also was used as an outcome measure. Cognitive development was measured by the Bayley Scales of Infant Development, Third Edition (BSID-III), which has been formally adopted into the Chinese language and environment and used in multiple studies across China [21]. Trained enumerators administered the BSID-III test, and standard testing materials and protocols were employed. Caregivers were present while the test was administered but were not allowed to assist the child in any way during the test. A child’s performance on a series of tasks, after being adjusted for by age in months and premature birth, determined BSID-III test scores.

Demographic characteristics consisted of two main categories: child characteristics and household characteristics. Child characteristics consisted of three main variables: gender of the child, whether the child had siblings, and whether the child was born prematurely. Household characteristics consisted of four main variables, including whether the mother was the primary caregiver (we did not collect information on early childhood education programs such as daycares or community nurseries, as few families in rural China send their young children to early education programs before age three [31]) mother’s age and education level, and whether the household was receiving social support through China’s Minimum Living Standard Guarantee program (a national welfare program for the lowest-income families nationwide, and an indicator of poverty in our sample).

#### 2.1.3. Statistical Analyses

We ran two sets of multivariate analyses in this study. The first set of analyses determined which child and household characteristics (if any) were associated with higher levels of book ownership, better early childhood reading practices, and relatively high levels of caregiver knowledge of and beliefs about early childhood reading practices. The second set of analyses determined whether reading practices and book ownership were associated with higher cognitive scores. All statistical analyses were performed using STATA 14.0, and we used STATA’s multiple linear regression model to conduct the multivariate analyses. We included the following variables as potential confounders in the multivariate analyses: child’s age, gender, and BSID-III cognitive score; whether the child was born prematurely; whether the child had siblings; whether the mother or grandmother was the primary caregiver; maternal age and educational level; and whether the household accepted Minimum Living Standard Guarantee payments.

### 2.2. Qualitative Research

To gain a more detailed, naturalistic understanding of the perspectives of the caregivers and factors underlying EECRP in rural China, a subset of the primary caregivers from the quantitative sample described above were selected for follow-up qualitative research. A total of 60 primary caregivers with children aged 10 to 30 months old were chosen to participate semi-structured qualitative interviews and field observations inside the home in 2017. Of these, 14 caregivers participated in a second set of follow-up interviews in 2020. All interview respondents came from two of the study counties (described below), from which six sample villages were randomly selected.

#### 2.2.1. Hypotheses

The purpose of the qualitative research was to gain a clearer understanding of why such a large share of caregivers from rural China did not buy or read books to their children even though many claimed that they knew about the importance of book ownership and reading and believed that they knew how to read to their children. To this end, we conducted interviews with primary caregivers and collected in-the-field observations to examine the specific constraints on book ownership and reading, including factors identified in our quantitative findings as associated with lower levels of book ownership and reading in rural China. As a first step in trying to meet this objective, we generated ex ante hypotheses before we started the collection of the qualitative data.

Using a social science framework that acknowledges that behaviors are, in part, shaped by constraints that individuals/households face, we formulate five hypotheses in consideration of factors that may act as barriers to the supply and demand of reading materials and practices. Specifically, our first hypothesis focuses on supply-side factors. This hypothesis assumes that many rural caregivers know that reading is important but are unable to use books to read to their children due to a lack of available, age-appropriate books for young children.

The next four hypotheses assume that there are no supply-side constraints (i.e., that books are available) but that there may be specific demand-side constraints that prevent caregivers from buying or using books to read with children. Specifically, in the second hypothesis (the first demand-side hypothesis), we state that even if caregivers have access to children’s books in their community, it could be that they are not reading to their children because they are unable to afford books. Due to low levels of income in many rural communities [32], caregivers may take into consideration available financial resources as well as the demand for other goods when they decide to purchase any specific item.

In the third hypothesis (the second demand-side hypothesis), we assume that books are available and income resources are sufficient but that rural caregivers are not reading to their children because they face difficult trade-offs regarding time use [33]. In other words, time constraints may be especially acute in rural households, and it may be an absence of sufficient time that is undermining the reading time with their children. Time constraints can come from a variety of sources. For example, it may be that one or both parents have out-migrated, which is a common situation in rural China [34], and the surrogate caregiver (usually the paternal grandmother) may be busy with tasks that leave her no time to read with the child. It also might be the case that there are other family members at home, e.g., other siblings or an elderly parent or grandparent, who might be taking up a large share of a caregiver’s time and energy, as women in rural China often have multiple responsibilities [13,35]. If this is the case, despite their ability to buy books that are available, caregivers may simply not have enough time to read to their children.

In the fourth hypothesis (the third demand-side hypothesis), after assuming there are no supply, financial, or time constraints, we consider the possibility that caregivers from rural China may have different aspirations for their children’s future that might not involve or prioritize reading skills. There are two factors that may influence this decision-making approach. First, it is well known that China’s education system is extremely competitive, within which rural children are systemically left behind in learning [13,22,23,36,37]. Hence, it might be that caregivers already have determined that their child may never be competitive enough to work his or her way up through the system, no matter what inputs (for example, book reading) they receive when young. Second, as the literature demonstrates that maternal subjective beliefs of their children’s skill formation predict their parental investment [38], it may be that the caregivers in rural China still believe that children from the countryside will almost all become farmers or unskilled laborers in the future. As such, it may be that they prioritize the development of skills (e.g., motor skills because they want a strong child) other than cognitive ones, which are promoted by reading, among other activities.

Finally (the fifth hypothesis and fourth demand-side hypothesis), we assume that books are available; caregivers have sufficient income and time to be able to read to their children; and caregivers have high educational and career aspirations for their children. In this scenario, however, we hypothesize that caregivers in rural areas might not read to their young children because they may not have sufficient knowledge about what to read to children or how to effectively read with them. It is also possible that caregivers believe they are reading effectively with their children, but, in fact, they may not have a true understanding of interactive parenting and EECRP. The literature shows that the educational levels of many caregivers, which are correlated with the quality of interactive parenting inputs in rural areas, remain low (less than one-third of the mother/grandmother caregivers attended high school; [21]). Low levels of formal education may limit the ability of caregivers to find, digest, and use information on effective ways to read. Given that 80% of our own sample of caregivers did not attend high school, caregivers in these areas may be particularly susceptible to misinformation about how, when to start, and what to read to their child.

#### 2.2.2. Interviews and Observations

The goal of the qualitative interviews and observations was to examine, through the voices of the caregivers themselves, the five hypothesized factors that may be keeping caregivers in rural China from either buying books or reading to their children. To achieve this goal, 30- to 90-min semi-structured interviews were conducted in 2017 and 2020 and included the following sections: (a) book ownership and usage at home; (b) household income and time use; (c) educational and career aspirations of caregivers for their children; and (d) actual reading practices (or lack of) with their children (e.g., whether caregivers read with their children as well as why, when, how, and where).

For the qualitative part of the study, the research team followed a standard protocol. All interviews were conducted one-on-one in Mandarin Chinese or local dialects in the homes of the respondents. During the interviews in 2017, detailed field notes were taken by a research team trained in qualitative interview methods. During the follow-up interviews in 2020, questions were added at the end to ask the respondents to express what they thought were the major barriers that restricted them from buying and reading books for their child during their child’s first three years. The interviews in 2020 were audio-recorded, transcribed, and translated into English.

In addition, to better test the first hypothesis (the supply constraint) and the fifth hypothesis (the knowledge-based demand constraint), field observations were conducted in 2017 in villages and nearby towns to determine whether there were any local bookstores, and if so, the types of books available, and inside homes of the caregivers to determine what books and reading practices were provided by caregivers for their children/grandchildren. Field notes were taken during the observations in villages/townships, and the observations inside the homes of caregivers were video recorded, transcribed, and translated into English by our research team. These data were collected as a means to assess the validity of the interview data.

#### 2.2.3. Qualitative Data Analyses

The analytical process was conducted in three stages. First, a method of de-identification (offering each participant an identification code of the interview and observation data), whether in the form of field notes or audio/video recording transcripts, was used to protect privacy. Second, a coding protocol was followed to organize, group, and categorize the de-identified interview and observational data into general themes in accordance with the five hypotheses. The research team looked for sentences or words across the field notes and transcripts that could address specific constraints that caregivers faced regarding book ownership and reading with their children. To assess the significance of the specific hypothesized constraint among the participants, the research team also counted the number of respondents who addressed the same theme. Finally, in the third step of the analysis, the categorized qualitative data were linked with the relevant quantitative results for a mixed-method analysis.

## 3. Results

### 3.1. Quantitative Findings

Table 1 presents the basic demographic characteristics of our sample children and corresponding households. The sample infants ranged in age from 6–24 months, with 30% of infants between 6–11 months (Row 2), 37% between 12–17 months (Row 3), and 33% between 18–24 months (Row 4). A slight majority of children in our sample were male (52%) (Row 5). About half of the children had siblings (50%; Row 6), and 5% were premature (Row 7). In terms of household characteristics, mothers were the primary caregivers in 70% of households (Row 8), with grandmothers as the primary caregiver for nearly all remaining households. Most mothers in the sample (65%) were above 25 years old (Row 9, Column 2), and 79% of mothers had a middle school education or lower (Row 10). In addition, 11% of households received Minimum Living Standard Guarantee welfare payments (Row 11), suggesting that this proportion included relatively (compared to nearly all households in the sample, which can only be described as low-income) low-income households.

#### 3.1.1. Prevalence of Book Ownership, Reading Practices, and Caregiver Perceptions

The rates of book ownership and reading practices are shown in Table 2. Levels of book ownership were found to be low, with the majority of households (59%) having no children’s books (Row 2). About 17% of households reported having one or two children’s books (Row 3), 13% reported having three to five children’s books (Row 4), and only 11% of households had six or more children’s books (Row 5). The results in the table also show that reading practices in rural China were uncommon. Only 21% of caregivers reported that they had read a book to their child in the last three days (Row 7), and only 18% had told their child a story over the same period of time (Row 6). Given the low rates of book ownership and the small share of caregivers who were reading to their young children, we would expect that EECRP rates would be even lower in rural China.

Table 3 shows that, despite these low rates of book ownership and poor reading practices, the majority of caregivers generally perceived that reading was important and believed that they knew how to read to their child. Of the caregivers, 72% indicated that they believed that reading to their child was relatively important (Row 1). Similarly, when asked how well they knew how to read to their child, 57% of caregivers responded that they were relatively sure that they knew (Row 3). This creates a seeming contradiction: The caregivers overall claimed they perceived reading to be important and claimed to know how to read to their child, yet very few did. (For the full distribution of responses, see Supplementary Table S1.)

#### 3.1.2. Correlates of Book Ownership, Reading Practices, and Caregiver Perceptions

Table 4 shows the results of the multivariate regression analysis of factors correlated with EECRP in rural China. The findings demonstrate that there are several statistically significant correlates. First, the identity of the primary caregiver is positively correlated with both book ownership and reading practices. Columns 1–3 (Row 4) show that when the mother (as opposed to the grandmother) was the primary caregiver, the household was more likely to own children’s books (0.05, *p* < 0.05). Mothers, as primary caregivers, were also more likely to read to their children (0.10, *p* < 0.01) and to tell stories (0.05, *p* < 0.05). In addition, mothers perceived reading as more important, relative to other caregivers (0.05, *p* < 0.1; Row 4, Column 4). Mothers also felt more confident in their knowledge of how to read to children, relative to other caregivers (0.20, *p* < 0.01; Row 4, Column 5).

The results in Table 4 (Row 5) also suggest that maternal age is a relevant factor for book ownership, reading practices, and caregiver perceptions. For maternal age, the results show that when mothers were older (above 25), the household was more likely to own children’s books (0.06, *p* < 0.05; Column 1). Older mothers also were more likely to read to their children (0.07, *p* < 0.01), but no correlation was found between maternal age and storytelling rates (Columns 2 and 3). Similarly, older mothers were more likely to perceive the importance of reading (0.07, *p* < 0.05), but no relationship was found between maternal age and confidence in reading knowledge (Columns 4 and 5). These results show that mothers as caregivers and older mothers were more likely to provide better early reading practices to their children.

Maternal education also was found to be correlated with book ownership, reading practices, and caregiver perceptions (Table 4, Row 6). When maternal education was low (having attended only middle school or lower), the household was less likely to own children’s books (0.13, *p* < 0.01; Column 1). Children whose mothers had low levels of education were also less likely to have been read to by their caregiver (−0.11, *p* < 0.01) and less likely to have had a story told to them (−0.10, *p* < 0.01; Columns 2 and 3). Low maternal education was also correlated with lower caregiver perceptions: Caregivers were less likely to perceive reading as important (−0.11, *p* < 0.01) and felt less knowledgeable about how to read to their children (−0.10, *p* < 0.01; Columns 4 and 5). In summary, from the multivariate analysis, it is clear that being an older, more educated mother is correlated with improved reading practices and increased book ownership.

The results in Table 4 (Row 7) also show that welfare and the number of children affected book ownership and storytelling rates, suggesting that there may be some material or temporal tradeoffs associated with EECRP. If a household received welfare in the form of Minimum Living Standard Guarantees (indicative of their facing severe financial difficulties), there was a decreaded likelihood of having children’s books in the household (−0.06; *p* < 0.1; Column 1). We found, however, that welfare had no statistically significant effect on any other variables. One interpretation of this is that lower incomes might be expected to affect the ability of a household to purchase books but might not necessarily affect other factors, such as caregiver perceptions.

The results also show the potential effect of time constraints on EECRP. According to the findings (Table 4, Row 2, Column 3), when a young child had (a typically older) sibling, this was associated with fewer books being read to them (−0.04; *p* < 0.05). To the extent that having more than one child reduces the time that a caregiver can provide to the younger child, this suggests that time limitations are constraining EECRP in rural China. Importantly, however, although the coefficient on the variable that measures whether a child had siblings was negative and statistically significant, its magnitude was small.

#### 3.1.3. Relationship between Reading Practices and Cognitive Development

Our second set of regression results (Table 5) shows that book reading, and book ownership were both related to higher cognitive development. Columns 1, 3, and 5 present the unadjusted correlations without controlling for demographic characteristics. Columns 2, 4 and 6 adjust for a set of demographic control variables (child gender, siblings, premature birth, whether mother is the primary caregiver, mother’s age, maternal education, and whether the family receives Minimum Living Standard guarantee payments). Looking first at book ownership, the unadjusted correlation shows that the household owning children’s books was associated with increases in cognitive scores by 1.56 points (*p* < 0.05; Row 1, Column 1). When we add the demographic control variables to the regression, the coefficient for owning children’s books remains significant at 1.43 (*p* < 0.05; Row 1, Column 2). Reading books to children was also shown to have a large, positive, and statistically significant relationship to child cognition: children whose caregivers who read to them had higher cognitive scores by 1.19 points (*p* < 0.1; Row 3, Column 5). Once demographic variables were added into the regression, however, this connection became insignificant. Nonetheless, these findings are consistent with the existing literature on the positive connection between early childhood book reading and cognitive development. Based on the results, it appears safe to conclude that early childhood reading is associated with higher levels of child cognition.

### 3.2. Qualitative Results

Thus far, the quantitative data have revealed that book ownership and reading practices were positively correlated with cognitive development in children and that the majority of caregivers perceived the importance of book ownership and reading practices and claimed that they understood how to read to their children. We also found, however, that rates of book ownership and reading practices were significantly low. With regard to the correlates of book ownership and reading practices, the results show consistently that the number of children in the household and lack of family wealth (as represented by Minimum Living Standard Guarantee payments) are negatively associated with rates of reading and ownership. In contrast, the identity of the primary caregiver (mother), maternal age, and education level are positively associated with reading and book ownership rates.

In this case, the quantitative results reveal an obvious contradiction: Caregivers perceived that books and reading are important and claimed that they knew how to read, yet the rates of book ownership and reading were low. Such a contradiction, of course, leads us to ask why this could be the case. Were there one or more constraints that kept caregivers and others in the households from buying books and reading to their children, even though they knew this was important? Is it possible that, even though caregivers claimed that they knew how to read, they were actually not knowledgeable about reading and its importance in developmental outcomes? To answer these questions, we examined the five hypothesized factors (described above) that may have limited the ability of caregivers to provide such important parental investment. The following section present the results of our qualitative interviews and observational data for a mixed-methods analysis to better understand reading practices between caregivers and young children in rural China.

#### 3.2.1. Hypothesis 1 (Supply-Side): Limited Access to Books

The first hypothesis, which concerns why so few caregivers read to their young children, is a supply-side argument. Some caregivers in rural China may want to read to their young children but may not be able to due to the lack of access to books that are suitable for young children. In other words, caregivers do not read to their children because there are no books to read.

To determine whether there is validity to this first hypothesis, we examine whether books are available from several perspectives. First, we rely on our observations and quotes from the qualitative interviews to understand whether books for children are available in the immediate vicinity of the homes of caregivers (that is, in local villages or townships). Next, we ask caregivers who reported having books at home about how they accessed these books and whether these approaches could provide access to most or all caregivers. Finally, we identify the location nearest most caregivers in which books for children are available and assess whether caregivers often visit those localities, making it possible that they, indeed, could have purchased books had they believed it was important enough (and if they had the resources to do so, which we address later).

According to our findings, if access is defined as books being available in the immediate locality of the residence of caregivers, it appears that book access might be considered an issue for many households. The field observation data suggest that there is almost no easy access to books in any of the villages or towns of sample caregivers. When our research team visited the villages or towns of the qualitative respondents, members of the team did not find a formal bookstore or any other store that sold children’s books.

In fact, when we asked about the accessibility of books inside the village of the interviewees, the responses from our qualitative interviews validated our observations.

“*(We) have not bought books for her…There is no bookstore in the village. You cannot buy books in the village*.”

(Grandmother, G−6977)

“*We do not have a bookstore in our village*.”

(Mother, M-4188)

“*There are no bookstores in our village*.”

(Grandmother, G-2664)

In addition to observations made in the villages themselves, the research team investigated larger stores or supermarkets in towns closest to the villages and found, in a few towns, that there were sometimes books for sale. Unfortunately, even in those stores, there were only magazines, comic books, and fiction novels for adults and, at most, a few exercise books for school-aged children. The qualitative research team did not find any age-appropriate children’s books in any of the sample towns that the research team visited. Based on these observations and the initial comments of the interviewees (and assuming access is defined as a book being available in the immediate vicinity of households), book ownership and reading practices may have been affected by local supply constraints.

When we continued the qualitative interviews and looked deeper into the data, however, we saw ways to access books. Indeed, if the absence of books in a household’s village or town were an absolute binding constraint, there would have been no households with books. Yet, the quantitative data showed that households generally owned an average of one book.

So, where did these books come from? According to caregivers in households that owned books, the books came from several sources. A number of respondents told us that the books in their home came from family members who worked in larger, urban areas and had brought the books back to the homes of the children.

“*We have a family member in Henan who brings us books sometimes. He knows where to get them, and which ones are good*.”

(Mother, M-9537)

“*Her mother and father work in Xi’an and they brought those books back home*.”

(Grandmother, G-2664)

“*Her father was in Xi’an and can buy books for her*.”

(Mother, M-4188)

Other respondents told us that online shopping was another way to access books.

“*We also bought books online*.”

(Mother, M-5954)

“*Those [children’s] books [over there on the mantel] were bought online*.”

(Mother, M-2937)

“*We get our books through online delivery. You buy [books], and they deliver them to you… It is not difficult to buy books [online]*.”

(Father, F-3988)

Although, in these cases, access to books did not appear to be a problem for a subset of households, this does not mean that these alternative sources of books were viable solutions for all households. For example, one cannot expect all mothers or grandmothers, even ones who know that books and reading are important, to have a relative or family member in a large city or to be able to travel to those distant cities to get books. In addition, not all caregivers (particularly, illiterate grandmothers) know how to order books online.

“*You see now the Internet develops so much; for example, there are lots of stuff on cellphones, and young people know everything. But I find that I do not know how to shop online, and I do not use Alipay either when I go out to buy things*.”

(Grandmother, G-2102)

“*I never bought [a book] for her … We (grandparents) are illiterate*.”

(Grandmother, G-0769)

In the interviews, we also were told that express delivery services in many cases would not bring packages to homes in many rural villages or even to towns in more remote mountainous areas. Hence, for those caregivers who did not have relatives in large cities, as well as those who did not know how to order books online and those who were living in remote villages with limited internet access, access to books could still have been a problem.

Although there were barriers that created some difficulty with access for rural caregivers, there was another source of books that was universally accessible to almost every household in the sample. According to the observations of the research team, in the county seats in every sample county (*n* = 22) that the research team visited, there were multiple stores that sold various books for young children. When respondents were asked about book access at the county level, their responses validated our observations that almost all county seats have bookstores.

“*We do not have places to buy books in our village, but we can buy books from the county. It is only about 25 to 30 km from here. It is convenient to take a bus*.”

(Grandmother, G-5620)

“*[Those books] were bought at the county bookstore…It was not difficult [to buy them]. It only takes 40 min by bus [to get to the county]. We usually go about once a month.*”

(Mother, M-7834)

“*There is a bookstore in the county... It is really easy to get there by bus. The bus runs every day, and one comes by every 10 or 20 min. It only takes about 20 min or less to get there. I generally go to the county every weekend, sometimes twice a week if I have things to do there*.”

(Mother, M-0460)

Even though not all villages have convenient access to the county seat, every one of our respondents reported that they were able to get to the county seat. In fact, many caregivers reported taking their child to the county either for essential health care or simply to run some errands.

“*When [my granddaughter] gets sick, we take her to the pediatric ward of the county hospital to see a doctor*.”

(Grandmother, G-0769)

“*When the weather is nice, I take [my grandson] to run errands in the county*.”

(Grandmother, G-5620)

“*Her physical health is poor and [she] needs to see a doctor five to six times a year. During every season [that is, every two to three months], [my daughter] needs to go to the hospital [which is in the county]*.”

(Mother, M-5954)

Although it is less convenient than if there were a bookstore in every village or town, it was possible to purchase children’s books in every county seat. Thus, access to books (though sometimes inconvenient) was not a fundamental barrier to EECRP in China. Hence, other factors must be limiting EECRP in a more fundamental way.

#### 3.2.2. Hypothesis 2 (Demand-Side): Financial Constraints

Assuming that all households are able to access books, which our findings above suggest, there must be something on the demand side that is keeping households from buying and reading books to their young children. Thus, we examine the first demand-side hypothesis: Caregivers do not read to their children because they cannot afford books.

To test this hypothesis, we initially asked respondents about their family’s financial condition. Next, we asked the respondents who stated that their households were not in absolute poverty whether they were willing to invest in their children. If they said that they were willing to invest in their children, we then asked directly whether the household did not buy books because they could not afford them. Finally, we explored what type of expenditures were actually being made by the household on their children. The logic of this final question is that, if a household was able to purchase other items for their children, then the household should be able to buy books if they prioritized reading as highly as they did those other items.

The interview data suggest that rural families, although not in extreme poverty, still have low levels of income. In general, respondents reported in the qualitative interviews that their lives as “rural people” were “not too bad and not too good,” and most families, indeed, had tight budgets.

“*We are not wealthy. We are all farmers. My grandson’s father works outside to feed all our family*.”

(Grandmother, G-6977)

“*When we earn more, we spend more. When we earn less, we spend less. So, [our financial condition] is not too good. But it also is not too bad*.”

(Grandmother, G-2102)

“*[We of course have a] tight [budget]. Her older brother needs to go to school, her grandfather is getting old, and her father works outside...We are always facing a tight budget*.”

(Mother, M-4188)

In fact, these responses were common to nearly all of the interviewees, as there were no well-off households in our sample. At the same time, however, most interviewed caregivers reported that their earning were certainly enough to cover the family’s daily expenses. This conclusion was supported by the quantitative results, whereby only 11% of households were receiving welfare payments. In other words, most households in the sample did not appear to face severe financial barriers.

Indeed, this conclusion is supported by additional comments from the interviewees. When asked how they felt about their current lives and compared themselves to the past, many respondents reported that either their own situations had changed for the better or that they believed their financial and living conditions had improved compared to older generations.

“*While no one would call our financial condition “good,” it is much better than when I was young*.”

(Grandmother, G-0769)

“*Compared to the previous generations, our life is better. If you work hard outside, you will make more money*.”

(Grandmother, G-2664)

“*Certainly, our lives now are better than the past*.”

(Mother, M-4188)

Once it was established that respondents believed that their households were better off than before, we asked about their ability and willingness to invest in their children, in general (without specifically asking about books or reading). Nearly all caregivers expressed that they are able and willing to invest more in their children so that their children would be able to grow up with more resources than in the past.

“*Rural people’s lives now, compared to the past, have improved… Now earning money is still hard, but if the hard work does not kill you, you can work harder, so you can keep making progress…After all, it is all for a better life, for our child, and for our family*.”

(Grandmother, G-2102)

“*The conditions in which we lived were very difficult; we did not have a lot. Now that we have more money and resources, I just want my child to grow up with more than I had*.”

(Father, F-3988)

“*We continue to work hard for our child’s future... Now we can wait a while before buying a house. As long as we can support our child’s education, we can buy a house later.*”

(Mother, M-9668)

“*Certainly, our child is the most important. As long as it is good for my child, I can spend less on myself and buy less clothing for myself*.”

(Mother, M-0460)

Based on these interviews, we concluded that, although households did have financial difficulties, caregivers were generally willing and able to invest in their young children.

Despite this improved financial situation and the willingness to invest in the development of their children, each household owned an average of only one book. When asked whether they believed that books were too expensive to afford, almost all respondents reported that buying books for their child was not too expensive.

“*Buying books is not expensive. One book is about ten yuan, and it is not difficult (financially) for us to buy books.*”

(Mother, M-7834)

“*Books are not expensive. Approximately six, ten, or eight yuan for a book. It was not difficult*.”

(Mother, M-6289)

“*It is not expensive. There are books that are more than ten yuan as well as those for a few yuan. That is a small amount of money*.”

(Grandmother, G-5620)

These statements indicate that financial constraints were not a barrier to book ownership for the rural households. The responses, however, also reveal a contradiction: Most caregivers seemed willing to invest in their child and to be able to afford books but still did not buy books or bought very few books.

In fact, in further discussion of their willingness to invest in their children, it became clear that the absence of books and reading in rural China was not due to the poverty of the families. This was shown in the caregivers’ report of their expenses on their child. Almost all respondents reported that buying baby formula for their child was the largest expense for their child even when the child was older (2 to 3 years old).

“*He drank baby formula from birth until he was 3 years old... [We spent] more than two thousand yuan a month for his baby formula*.”

(Grandmother, G-6977)

“Interviewer: *What was the biggest expense before your child was 3?*

Respondent: *Expenses for baby formula.”*

(Father, F-3988)

According to our qualitative interviews, rural households spent non-trivial shares of income on baby formula, which is consistent with previous studies that show high consumption rates of infant formula among households in rural China [39,40].

So why did families spend so much on formula? When we asked, nearly all respondents explained that high spending on baby formula was due to their willingness to spend money on things that were important to raising a healthy child.

“*We spent most on baby formula [before she was 3 years old]… To have our child grow her body, we wanted her to have more baby formula. It was very expensive.*”

(Mother, M-4188)

“*For our rural people, we want our child to be healthy, eat well, and drink well*.”

(Grandmother, G-7728)

Importantly, the large share of spending on baby formula, while perhaps needed for some younger children (under 12 months) may represent an unnecessary expense for older children (12 to 30 months old). Although a discussion of infant formula use is beyond the scope of this paper, the main point is that such expenditures indicate a willingness and ability of most caregivers to invest in their children when they believe it is important for their child’s development.

Based on the findings and the associated logic, Hypothesis 2 is not supported. In other words, financial constraints do not limit most families from buying and reading books to their young children. There are clearly at least some fungible resources in most families, yet, in the case of most households, caregivers were not prioritizing the purchase of books for their young children.

#### 3.2.3. Hypothesis 3 (Demand-Side): Time Constraints

We investigate whether time constraint is a demand-side barrier that prevents caregivers from reading to their young children. The need to address this issue becomes even more important after we have shown that, for most caregivers, the absence of books at home and the low level of reading to their children is not due to either supply-side constraints or financial constraints on households. Acknowledging that these constraints are not barriers, we wonder whether caregivers are not reading to their children because they do not have the time to do so.

We assess this time-constraint hypothesis by first asking caregivers about their household duties as a way to assess their daily routine. If caretakers reported that their daily routines were busy, we then asked questions to ascertain how much flexibility caretakers had regarding their schedules—whether caregivers had a lot of tasks but had some flexibility in those tasks (as defined by having other family members help with their tasks or having time for certain non-urgent tasks) and whether it was possible for them to “make” time to read to their children. Finally, we asked caregivers directly whether they felt that the reason they did not read to their children is that they believed that they did not have enough time.

The interview data show that many caregivers do have busy daily routines with multiple responsibilities at home. In our conversations, caregivers would mention having daily commitments that required them to spend significant amounts of time on housework and farm work. In some households, caregivers (both mothers and grandmothers) would refer to the time that they had to spend taking care of others in the household, such as grandparents (especially if they were sick) or other siblings (e.g., an older brother or sister in preschool or elementary school).

“*[I need to] wash clothes, cook meals, do housework… I am always very busy. I cook three meals every day; we also raise pigs and chickens*.”

(Grandmother G-0769)

“*I have little time to be with my daughter. I am taking care of my [farming activities] every day.*”

(Father, F-3988)

“*I need to cook three meals … do laundry every day and send my older grandson to school and back. I am really busy*.”

(Grandmother, G-2664)

“*There are always a lot of annoying chores at home… I have older grandparents to take care as well as the young child. The old grandparents are not in good health condition, and you must take care of them.*”

(Mother, M-9668)

These multiple responsibilities suggest that some caregivers might not have much time to read to their child. However, when the research team more carefully explored the daily activities of caregivers, many statements revealed that respondents, while often busy, did have some amount of time to engage in leisure activities, and some caregivers appeared to prioritize those activities over reading to their child.

“*[Although I] do housework… [I also have time] to exercise and play some music*.”

(Mother, M-6289)

“*[Although there are a lot of things to do around the house,] today’s young parents often sleep late at home and play with their cellphone all day and night, checking Tik Tok and Kwai videos. [My daughter-in-law] checks her cellphone, puts on her make-up, plays, travels around. She always has time to do these things, [and then says that she] does not have time to read to her child*.”

(Grandmother, G-2102)

“*Of course, almost [all rural caregivers] have time to chat with neighbors all day… But most people do not take more time to read with their child*.”

(Mother, M-4188)

In addition, even though most respondents were full-time caregivers, household dynamics are such that caregivers can (and do) spread out tasks among other family members. Previous studies show that many caregivers also lived with other secondary caregivers who helped them to take care of a child, and even those caregivers who also had to manage farm work often had other family members to help with these tasks [41,42]. Our interview responses are in keeping with the results of these studies:

“Interviewer: *When you are busy with farm work, are there other family members who help you to take care of the child?*

Respondent: *Yes, his grandfather and grandmother [help me]*.”

(Mother M-6289)

“*We have two children… [Their] grandmother is also at home… I do not have other things, and I am a full-time housewife, doing the same thing every day... We have a few crops to grow, but his grandmother does that, and I do not need to do it.*”

(Mother, M-0460)

In fact, some caregivers stated that, despite multiple responsibilities at home, they are willing to take time away from those other chores for their child.

“*Every day, I need to boil the feeding bottles and wash them. [My granddaughter] also drools a lot… I wash her clothes every day… But I can take the time to be with her and put all other things aside…In order to take care of her full time, we stopped our farm work, stopped raising pigs and chicken; even no rabbits at home*.”

(Grandmother, G-2102)

“*I do not have time during the day, but I do have time in the evening*.”

(Grandmother, G-2664)

Despite the fact that many of the caregivers stated that they were able to make time to read to their child, few did, as noted earlier. When our qualitative research team explicitly asked whether it was due to time, many stated it was not due to a lack of time.

“*Of course, it is important [to read to my grandson at home], but I do not read to him... It is not because of time and I have time*.”

(Grandmother, G-7728)

“Interviewer: *You just said it is important to read with your grandson, but you did not. Was it because you do not have time or any other reasons?*

Respondent: *My main job is to take care of the child... I have lots of time to be with him*.”

(Grandmother G-5620)

“*It is not because of time [that I do not read with him] because I am with him all of the day and night. [It is] mainly because sometimes I am in a bad mood for the little things of daily life*.”

(Mother, M-0460)

The statements above demonstrate that, in many cases, caregivers themselves did not believe that a lack of time kept them from reading to their child. These responses show that, although almost everyone was busy, caregivers either had time or could have made time to read to their child. Unfortunately, as we know from the quantitative data, most did not. Therefore, we believe that the time-constraint hypothesis is not supported. In short, most caregivers have the time to read to their young children but did not make it a priority.

#### 3.2.4. Hypothesis 4 (Demand-Side): Educational and Career Aspirations of Caregivers for Their Children

The findings show that access to books, ability to afford books, or availability of time did not fundamentally restrict EECRP in rural China. Here, we examine the aspirations of caregivers for their children. Specifically, we seek to determine whether low aspirations for their children’s education and career was one of the reasons that caregivers chose not to read to their young child. We assess this hypothesis about aspirations by first asking caregivers about their educational and career aspirations for their children. After caregivers reported their aspirations, we then asked them how they thought those aspirations could be achieved and how they, as caregivers, could help to prepare their children to achieve these goals.

The interview data show that most caregivers had high educational aspirations for their children. Most caregivers reported that, because they themselves did not have a chance to go to college, they hoped that their children would gain more educational opportunities that they felt they never received. As the following excerpts show, many caregivers wanted their child to go to the best university, and some even wanted them to get a graduate degree.

“*I hope [my granddaughter] can go to the best university … like Tsinghua University… I want her to be more successful in the future because our old generation has very little knowledge, and what we learned is very limited*.”

(Grandmother, G-2102)

“*I hope my two children can at least go to college because I was not able to go to college and feel very regretful, so I do not want them to repeat my regret.*”

(Mother, M-0460)

“*To be honest, for my generation, it is already too late to receive a good education. We had it hard, so we want our children to do better than we did, to have a better life*.”

(Mother, M-9668)

“*If my child gets a Ph.D. degree, it brings honor to our entire family. I want him to find a good job and become an entrepreneur*.”

(Mother, M-5954)

“*In today’s society, one cannot live without knowledge. Maybe because now we have less children than the past and we now, compared to the past, when we did not have enough food, can eat well and clothe well, so we have high expectations [for our child]*.”

(Mother, M-4188)

We found that these high educational aspirations for their children often centered on academic achievement in schools and were strongly motivated by concern about the child’s future occupations. As seen in the following excerpts, caregivers believed that, by doing well and studying hard in school, their children could find “better jobs,” which they defined as stable employment that was not physically exhausting, in contrast to the demanding manual labor available to those with little or no education.

“*I want him to study well so that he can get a stable job that does not require manual labor in the future*.”

(Mother, M-6549)

“*I told my grandson that, if he wants to work in an office instead of the farm, he must gain knowledge and a degree to work in an office. Every [grand]parent hopes their [grand]children can excel their parents*.”

(Grandmother, G-5620)

“*I hope he can study well in elementary school, middle school, and high school. I hope he can go to college, no matter which college. After college, no matter what jobs he finds, it is better than being a farmer. Farmers work by labor, but those who go to college do not*.”

(Grandmother, G-6977)

This indicates that many caregivers hope that their children can move beyond the manual, unskilled labor that most of these caregivers currently rely on.

When asked how to prepare their child to achieve these goals, many caregivers believed that they played a less important role when the child was young (before school age). They thought that it was the school’s responsibility and the children’s own ability to work hard in school when the child gets older.

“*This [high educational goals] can only be prepared by teachers in school and by [my son] working with the teacher; the teacher teaches him, and he listens to the teacher*.”

(Mother, M-6289)

“*When she grows up, it depends on her own ability to find [the] kinds of job to support herself and family. Really, it depends on herself*.”

(Grandmother, G-7728)

“*It depends her own efforts. For example, if she wants to go to college, she must study hard by herself*.”

(Mother, M-5954)

“*To help my son, I think [the] first thing is to take good care of him so that he can have a healthy body and take less medicine.... If he has difficulty to learn, I do not know how to teach him, but I can do my best to pay for tutorial class to let other teachers to help him … it really depends on himself if he wants to become successful in the future. It depends on himself*.”

(Mother, M-2937)

These excerpts show that caregivers rely on later schooling (or their child’s inherent ability or motivation), instead of early parenting behaviors, to actualize their high expectations. No caregiver explicitly expressed the idea that early childhood education and stimulation at home was an important part of a child’s human capital development process. This reliance on others suggests that, despite high expectations for their children, caregivers do not know (or do not believe that they know) how best to prepare their children to achieve these goals and are unaware how certain practices, such as early childhood reading, can help their children.

Given how caregivers rely on school education to prepare for their child’s success, we wanted to understand the caregivers’ perceptions of the quality of the local schools that their children will attend. When asked if their local education quality is good enough to prepare their children to meet their high aspirations, most caregivers did not believe so, as the following responses show:

“*On a whole, rural schools are worse than the city ones from all perspectives*.”

(Mother, M-6289)

“*Certainly, the education quality in the city is better than the schools here [rural areas]*.”

(Mother, M-0460)

“*[Local education quality is] sort of just so-so. It depends on the child’s own endeavor. If you study well, it does not matter which school you are in; similarly, even if the school is very good, but if you do not study, it also does not work... The education in the county is better than [in] our village, so it can prepare more students to go to college*.”

(Grandmother, G-7728)

“*Compared to the city, our rural education quality is relatively poor and lags behind. [Poor rural education quality] certainly cannot meet our expectation [for our granddaughter]… The education quality here is too poor... The quality there [in the city] is much better than our rural schools… There are very few [who] can go to college [from the local schools]. For example, during a year, we can count how many students can be prepared by our village schools to attend college, and that is only three to four… in our whole area*.”

(Grandmother, G-2102)

These responses show that, although caregivers view schools as the main way that their children can achieve their educational aspirations, caregivers know that local rural school quality is poor and certainly cannot well prepare their children for college or better jobs, a conclusion supported by previous studies [43,44].

Despite knowing that local schools are of low quality, caregivers did not appear to be discouraged but, rather, continue to work hard to support their children. We found that their support is in the form of saving as much as they can for the child’s future schooling or college, as caregivers believed that everything else is out of their hands.

“*When she is older, I will not have her to go to a local school, and I will do my best to put her in a city school…We support her by saving money for her future when she goes to college*.”

(Grandmother, G-2102)

“Interviewer: *How do you support your child to achieve the goal to go to college and get a Ph.D.?*

Respondent: *That is to financially support her to go to college.*

Interviewer: *Is there anything else that you have done to prepare her for these goals?*

Respondent: *No, I have not thought about it.*”

(Mother M-4188)

“*[Whether or not go to college] really depends on herself, and no one can help her… I will let her nature take its course*.”

(Mother, M-2937)

“*If other children can go to university, then she should also be able to go to college if she works hard*.”

(Grandmother, G-2664)

As the above excerpts show, caregiver efforts are focused mainly on the future, via savings and financial support, rather than on enriching early parenting practices, such as reading with their child now, at a younger age. The ability and willingness to invest in their children when they are older, even though the families recognize that their local schools are not the best, is evidence that low investment in early childhood education, including reading at home before schooling, is not due to the fact that they already have given up on their children’s chances but, rather, due to poor local schooling opportunities.

In summary, these findings demonstrate that there is an absence of early childhood preparation among rural caregivers despite their high expectations. Most caregivers have high aspirations for their children to pursue higher educational degrees and then to work at skilled labor, but they appear to not be aware that investing in children at home when they are young is part of an investment strategy that will enhance future human capital outcomes. Hence, we believe that the nature of caregiver aspirations is not a fundamental barrier to EECRP in rural China.

#### 3.2.5. Hypothesis 5 (Demand-Side): Knowledge Constraints

Because we determined that access to books, availability of resources (financial or time), and caregiver aspirations are not fundamental barriers to EECRP, we wondered whether it was possible that the limited knowledge of caregivers is the fundamental barrier to EECRP in rural China. Thus, we investigate whether the demand for reading materials and willingness for caregivers to spend time reading to their children is being undermined by the fact that their knowledge of the subject is insufficient or imperfect.

In investigating this demand-side hypothesis, we attempt to determine what caregivers actually know. Specifically, we seek to understand whether parents understand that reading is important to the development of their children during the first three-year critical period of infant and toddler development. We first investigate through our qualitative interviews why so many caregivers did not read to their child before the child was 3 years old, despite most caregivers saying that reading was relatively important. We then examine whether those few caregivers who did read to their child had a solid understanding of EECRP and used EECRP to read to their children. We then ask caregivers how they made decisions about early childhood reading (e.g., where they got their information) and then conclude by asking caregivers directly what they saw as major barriers that prevented them from reading to their children.

The quantitative results showed that most caregivers (80%) did not read to their young (0- to 3-year-old) child. When asked why, most caregivers reported that they believed that the optimal time to read to their child was later in the child’s life.

“*When my children can speak, they can understand things. Then I will read with them.*”

(Mother, M-6871)

“*He does not listen to what I say yet, so why would I read to him if he is not going to listen to me?*”

(Mother M-7834)

“*Why would I even spend time reading with my child who cannot identify characters yet? I would wait to read with him until he knows how to read*.”

(Mother M-2937)

These responses show that most caregivers who did not read to their child had little to no knowledge that reading to their child during the critical first three years is important to the cognitive development of the child.

In addition, some of those who did not read to their child misinterpreted some early childhood behaviors, such as a lack of physical coordination, as signs that their child was too young to be read to, as the following excerpts show:

“*Reading is important, but [she will] tear the pages up in the book after a while*.”

(Grandmother, G-0769)

“*I will buy books for him after he turns three because he would only tear the pages now*.”

(Mother M-7112)

“*Therefore, among most caregivers, the optimal time to start buying and/or reading books was erroneously postponed until the child was older than 3 or 4 years. When he starts preschool, then I will start buying books for him. But for now, I do not think he needs any*.”

(Grandmother G-0278)

“*They can start reading when they are starting preschool, around 3 or 4 years old*.”

(Grandmother G-7728)

“*Before age 3, she does not know how to read or speak. So, when she is old enough, [books] will be useful*.”

(Grandmother G-0769)

The quantitative results showed that 80% of caregivers believed that reading was important, yet they did not buy and/or read books with their child. The interview responses resolve this contradiction by showing that, due to a lack of knowledge about the importance of early childhood reading and its link with early childhood development, caregivers erroneously delay reading until after 3 years of age. In other words, caregivers believe that reading is important but do not read to their young children because they believe that reading is important only later in the life of their child. This indicates that knowledge is a high barrier for most caregivers and prevents them from providing EECRP to their young children.

Yet, as the quantitative data show, some caregivers (20%) did read to their child. We wondered whether knowledge was a barrier for them as well as what and how they read to their child. The interviews show that, even if some caregivers read to their child, many did not start reading until their child was 2 years old (which, though better than most caregivers, is still late) and used only object-labeling books, as the following excerpts reflect:

“*From when she was 2 years old [we first bought a book for her]… [We have] very few [books at home] … to let her identify fruits, identify mother, father, and aunty*.”

(Mother, M-4188)

“*I started reading to her after 2 years old…Basically, our books are those with pictures to identify objects like cars, food, and colors, and we did not have a storybook.*”

(Mother, M-0460)

“*[We have books], the kind of books [that teach how] to recognize fruits and vegetables*.”

(Grandmother, G-2102)

These interviews suggest that object-labeling books are often read to 2-year-old children. The literature shows that these object-labeling books are more suitable for younger children (under 12 months) but can, in fact, limit development in older children. This is because, due to the high pace of cognitive development during the first three years, the most effective reading materials vary for different age levels and using reading books that are not age-appropriate can slow the pace of childhood cognitive development [17,18]. In addition to the interview responses, our observations inside the homes of caregivers show 94% used object-labeling materials. Therefore, both interview and observation data show that most caregivers who read to their child had limited knowledge of which types of reading materials were age-appropriate and developmentally stimulating for their children.

Further, our field observations inside the home of these caregivers show that they used these object-labeling books exclusively to repeatedly quiz the child while expecting a “correct answer.” The following transcript of recorded observation shows a typical reading style among most of the caregivers whom we observed.

“Caregiver: *(Points to a hardcover object-labeling book.) What is this?*

Child: *Horse*.

Caregiver: *Right. What is this?*

Child: *Chicken*.

Caregiver: *Wrong! (again): What is this? This is a duck. How can it be a chicken?*

Child: *Duck*.

Caregiver: *Yes, right.*” (Observation Video Transcript).

This rote-learning reading style Stands in contrast to EECRP, which include reading practices that use age-appropriate books and interactive reading styles. EECRP actively engages the child in the reading process by, for example, asking the child to point to and touch the book and to interact with the reading materials by asking open-ended questions. Previous studies have shown that, during the first three years, interactive reading can lead to better brain development [1,7,16]. Yet, as we observed, most caregivers did not use age-appropriate materials, nor did they read to their child in an interactive way. Our observational data suggest that, despite most caregivers reporting that they felt “relatively sure” that they knew how to read to their child, very few caregivers actually knew how to engage in EECRP with their children. Thus, knowledge was still a barrier even for caregivers who did read to their child, which, in addition to others not reading to their children due to a lack of knowledge, means that knowledge barriers were an issue for almost all caregivers in rural China.

Given that almost all caregivers, regardless of whether they read to their child, seemed to have misconceptions about or did not properly conduct EECRP, our qualitative interview team asked caregivers where they got their information about reading. Most caregivers (75%) reported that they made their reading decisions based mainly on their intuition. Only a few received information from sources other than themselves, usually other caregivers.

“Interviewer: *Do you read to your grandson?*

Respondent: *He cannot read even if you read to him*.

Interviewer: *How do you know this?*

Respondent: *I do not know because I have not read to him…Based on my own intuition, [I] feel that he does not understand [the book]*.”

(Grandmother G-6977)

“*I just kind of know that these books work, based on my own understanding*.”

(Grandma, G-0769)

“*I saw another parent in the supermarket buying books for her child, who seemed to be around the same age as my son. That is why I started buying books for him*.”

(Mother, M-0493)

The fact that so many caregivers relied on intuition for early childhood reading information suggests a widespread lack of access to accurate knowledge of EECRP among most caregivers in rural communities.

Furthermore, at the end of our interviews, many caregivers remarked that they believed that their own lack of knowledge and literacy skills constituted a major barrier that kept them from making better reading decisions for their young (grand)child, as the following excepts show:

“*After all, because he is too young and does not understand anything…We always feel that we adults know too little and do not have much knowledge*.”

(Mother, M-4188)

“Interviewer: *Why did you not buy books for your grandchild?*

Respondent: *Because our rural people are illiterate… No one can read to her, so what do you buy it [a book] for?*

Interviewer: *Why did you not decide to buy and read books with your granddaughter?*

Respondent: *Just because I have never thought about it.*

Interviewer: *Why do people in rural communities not read with their young children?*

Respondent: *Because to be honest, rural people do not like to read*.”

(Grandmother G-7728)

“*I do not have that kind of knowledge… Sometimes in a book, I can only recognize a couple of words. How can I read to her?*”

(Grandmother, G-0769)

These responses confirmed our hypothesis that it is the inaccurate, inadequate knowledge of caregivers that constitutes the fundamental barrier to EECRP in rural China. Even though most caregivers believed that reading to their child was important, they were not aware that reading during the first three years is critically important to the development of their child; as such, most caregivers postponed buying and reading books to their child until pre-school age (after this critical period has passed). Even those few who read to their child, due to their inadequate or inaccurate knowledge of EECRP, they did not truly know what and how to read to their child, and, thus, their reading practice was inefficient. A lack of accurate information about EECRP allows such misconceptions to persist, and even the caregivers themselves recognized that their own lack of knowledge was affecting their reading practices. Thus, we feel confident in concluding that the widespread lack of knowledge about EECRP constitutes a fundamental barrier that is keeping caregivers rom providing timely, effective reading practices in rural China.

## 4. Discussion

Cognitive delays are unfortunately common in rural China, with nearly half of rural infants experiencing such delays [9,13,20,21]. Although many studies have suggested that these delays are due to an absence of interactive parenting (such as reading) during the first three-year critical period [13,22,23], few have specifically examined EECRP, its connections to cognition, and the factors that cause such low rates of EECRP during such a crucial period for children in rural China. To answer these questions, we conducted this mixed-methods study that combined the results from a quantitative survey conducted in rural northwestern China with 1748 observations across 100 villages/townships as well as an analysis of extensive interviews with 60 caregivers from these same study sites. Our quantitative data show that, despite significant associations between EECRP and higher cognitive development, and despite caregivers believing that reading is important and that they knew how to read to their children, the rates of EECRP were extremely low in our sample. This reveals a glaring contradiction: Most caregivers perceived the importance of book reading and reported they knew how to read, yet most did not buy or read books for their child before their turning 3 years old.

To understand this contradiction, we interviewed caregivers to examine five hypothesized factors, one supply-side (access to books) and four demand-side constraints (financial, time, caregiver aspirations for their children, and caregiver knowledge), that may prevent caregivers from providing EECRP for their children. Of all these factors, we found that it was not the supply of books, the absence of capital or time, or the low aspirations of caregivers that were behind the low levels of reading at home to children. Rather, it was the lack of knowledge of EECRP among caregivers that was fundamentally limiting EECRP in rural China. Although the caregivers reported results that they believed that reading was important and knew how to read to their children, most caregivers did not truly understand the importance of EECRP for cognitive development during the first three-year critical period and had multiple misunderstandings in regard to the importance of early stimulation.

The absence of EECRP, due to the limited knowledge of caregivers, in rural China is part of a broader issue of lack of stimulation among rural children that contributes to the abnormally high rates of cognitive delay. The international literature has shown that psychosocially stimulating parenting practices during the first three years of a child’s life (of which reading is one of many possible stimulating activities) is fundamental to the healthy development of children [45]. Yet, as our and other studies show, caregivers in rural China seldom conduct these types of activities [20,21]. The rate of reading (20%, as shown in the study) in rural China remains much lower when compared to urban China and rural areas in other developing countries, such as Columbia, where approximately 73% of rural caregivers read to their child [46]. Our findings about a lack of EECRP, therefore, reflect a broader lack of interactive parenting practices in rural China.

Our study is also consistent with past studies in identifying the lack of caregiver knowledge as the fundamental barrier to better parenting practices and, thus, increasing cognitive outcomes [13,20]. In addition to limiting interactive parenting practices, this lack of knowledge among caregivers has been shown to contribute to micronutrient-based cognitive delays, resulting in an abnormally high prevalence of anemia (42%) in rural areas and high rates of cognitive delay (49%) [13,21]. Unfortunately, it appears that such important caregiver knowledge has not been specifically taught or addressed in schools, communities, or public health systems in rural China. Therefore, unless action is taken quickly to improve the levels and quality of caregiver knowledge in rural China, these trends will likely persist. Further, this lack of knowledge among caregivers may potentially aggravate existing rural-urban inequalities in contemporary China, ultimately preventing rural citizens from participating equally in the educational and employment opportunities associated with economic growth [47,48].

The results of this study suggest that policymakers in China should increase investments in promoting early childhood development, particularly EECRP, in rural areas to increase the level of cognitive skills among rural children. Numerous studies have shown that programs that promote interactive parenting have positive, long-lasting impacts on developmental outcomes for younger children [2,49,50]. Recent research also has shown that such investments in early childhood development have high economic returns as compared to later investments at an older age [2,7], and several Latin American countries already have made large investments in ECD to raise human capital and promote economic development [51]. Studies conducted in rural China also have found ECD interventions to be effective in increasing the cognitive abilities of rural Chinese children [52], which may better prepare these children to achieve higher educational and career goals, potentially decreasing the urban-rural divide, and boosting the economy by producing more skilled laborers [20]. Given the rapid increase in in the rates of smartphone ownership and internet usage in rural China in recent years [53] and corresponding access to online book delivery, ECD interventions that target EECRP have the potential to dramatically improve reading skills among caregivers and children. It is our hope that future policymakers and researchers will build on the results of this study to further investigate how to deliver knowledge of EECRP for China’s rural caregivers as part of a broader policy focus on promoting ECD to decrease the rate of cognitive delays and, as a result, increase the proportion of skilled laborers for China’s future development.

## 5. Conclusions

This is the first study to conduct an in-depth analysis of the factors limiting rural caregivers in China from engaging effectively in stimulating parenting practices such as reading. Using mixed quantitative and qualitative methods, this study investigates the nuances in the self-reported knowledge and beliefs of caregivers of young children in rural China. We find that book ownership and reading books with children is associated with improved ECD, and most caregivers reported believing in the importance of reading and that they know how to read with children. Despite these assertions, however, the majority of households in our sample did not read with their children. This contradiction appears to be due to a lack of knowledge around effective early childhood reading practices (EECRP) and how to engage in EECRP with young children. These results shed new light on ECD and parenting in rural China, with implications for China’s future economic trajectory.

We acknowledge three limitations of this study. First, due to the cross-sectional nature of our quantitative data, we cannot ascertain causal relationships between EECRP and ECD with certainty. Second, because the normative sample of Bayley-III has not established yet in China, we rely on a reference population and norm from the United States. However, a broad array of literature in rural China has used a United States reference population to identify delays among Chinese children [21,54,55], suggesting that it is generally acceptable to do so. Finally, the quantitative and qualitative data were gathered from one region in China, and the results may not be representative of all rural communities across China. Future research should aim to quantitatively and qualitatively examine EECRP in rural communities across China to confirm these findings and identify regional differences in parental investment among rural caregivers.

## Figures and Tables

**Table 1:** Basic characteristics of children and caregivers (*n* = 1748).

Characteristics	*n* (%)
Panel A: Child Characteristics	
(1) Infant Age at time of survey	
(2) 6–11 months	532 (30%)
(3) 12–17 months	645 (37%)
(4) 18–24 months	571 (33%)
(5) Infant is male	908 (52%)
(6) Infant has siblings	873 (50%)
(7) Premature birth	82 (5%)
Panel B: Household Characteristics	
(8) Mother is primary caregiver	1219 (70%)
(9) Mother is older than 25 years at time of survey	1140 (65%)
(10) Mother has a middle school education or below	1383 (79%)
(11) Family receives Minimum Living Standard Guarantee	192 (11%)

Data Source: Authors’ survey. Note: (i) Data are presented as frequency and percentage. (ii) The sample size *n* = 1748 refers to 1748 caregiver–child dyads included in the study.

**Table 2:** Book ownership and reading behavior among caregivers (*n* = 1748).

Variable	*n* (%)
(1) How many children’s books do you have?	
(2) 0 children books	1027 (59%)
(3) 1–2 children books	303 (17%)
(4) 3–5 children books	226 (13%)
(5) 6 children books or more	192 (11%)
(6) Told story to child in last 3 days	318 (18%)
(7) Read book to child in last 3 days	365 (21%)

Data Source: Authors’ survey. Note: (i) Data are presented as frequency and percentage. (ii) The sample size *n* = 1748 refers to 1748 caregiver–child dyads included in the study.

**Table 3:** Perceived importance and knowledge of reading practices among caregivers (*n* = 1748).

Variable	*n* (%)
Perceived importance of reading to child (1 = Totally unimportant, 7 = Extremely important)	
(1) Relatively important (5–7)	917 (72%)
(2) Relatively unimportant (1–3)	352 (28%)
Do you know how to read to your child? (1 = Not at all, 7 = Absolutely)	
(3) Relatively sure (5–7)	812 (57%)
(4) Relatively unsure (1–3)	601 (43%)

Data Source: Authors’ survey. Note: (i) The sample size *n* = 1748 refers to 1748 caregiver–child dyads included in the study. (ii) The answer choices followed a 7-point Likert scale, with 1 being “unimportant” or “not knowledgeable at all” and 7 being “extremely important” or “absolutely knowledgeable”. For ease of analysis, the responses are placed into two groups: relatively positive and relatively negative. The response of 4 is excluded from both categories, as it may represent a neutral opinion. The distribution of responses on the full 7-point Likert scale is provided in Supplementary Table S1.

**Table 4:** Correlates of book ownership, reading behavior, perceived importance and knowledge of reading practices (*n* = 1748).

	Owned Children’s Books	Told Story to Child	Read Book to Child	Perceived Reading as Important	Confident in Knowledge of Reading
	(1)	(2)	(3)	(4)	(5)
(1) Infant is male	−0.01	−0.01	−0.02	0.00	0.02
	(0.02)	(0.02)	(0.02)	(0.03)	(0.02)
(2) Infant has siblings	0.09 ***	−0.03	−0.04 *	−0.01	−0.02
	(0.03)	(0.02)	(0.02)	(0.03)	(0.03)
(3) Premature birth	−0.05	0.00	−0.03	−0.02	−0.06
	(0.06)	(0.05)	(0.05)	(0.06)	(0.07)
(4) Mother is primary caregiver	0.05 **	0.05 **	0.10 ***	0.05 *	0.20 ***
	(0.02)	(0.02)	(0.02)	(0.03)	(0.03)
(5) Mother older than 25 years	0.06 **	0.02	0.07 ***	0.07 **	−0.00
	(0.03)	(0.02)	(0.02)	(0.03)	(0.03)
(6) Mother has middle school education or below	−0.13 ***	−0.10 ***	−0.11 ***	−0.11 ***	−0.10 ***
	(0.03)	(0.03)	(0.03)	(0.03)	(0.03)
(7) Family receives Minimum Living Standard Guarantee	−0.06 *	0.02	−0.01	0.01	−0.06
	(0.03)	(0.03)	(0.03)	(0.04)	(0.05)

Data Source: Authors’ survey. Note: (i) The sample size *n* = 1748 refers to 1748 caregiver–child dyads included in the study. (ii) Table 2 shows the distribution of the number of children’s books owned by sample households. For ease of analysis, the responses were placed into two groups: owns children’s books (41%) and no children’s books (59%). (iii) A 7-point Likert scale was used to measure the perceptions of the importance of and knowledge about early childhood reading. Caregivers with response scores of 5–7 are considered to perceive reading as important or have confidence in reading knowledge. The distribution of responses on the full 7-point Likert scale is provided in Supplementary Table S1. (iv) OLS estimates are reported in the table, and robust standard errors clustered at the village level are presented in parentheses. (v) *** *p* < 0.01, ** *p* < 0.05, * *p* < 0.1.

**Table 5:** Correlation of book ownership/reading behavior and cognitive development (*n* = 1748).

Dependent Variable	BSID-III Cognitive Scale Score					
	(1)	(2)	(3)	(4)	(5)	(6)
(1) Family owns children’s books	1.56 **	1.43 **				
	(0.67)	(0.69)				
(2) Told story to child			0.64	0.40		
			(0.76)	(0.77)		
(3) Read book to child					1.19 *	0.92
					(0.64)	(0.66)
(4) Child and Household Characteristics	No	Yes	No	Yes	No	Yes

Data Source: Authors’ survey. Note: (i) The sample size *n* = 1748 refers to 1748 caregiver–child dyads included in the study. (ii) Row (4) indicates whether the analysis controls child and household characteristics. Columns (1), (3) and (5) present the unadjusted correlations without controlling for demographic characteristics. Columns (2), (4) and (6) adjust for a set of demographic control variables, including child gender, whether the child has siblings, premature birth, whether the mother is the primary caregiver, mother’s age and education level, and whether the household was receiving financial support through China’s Minimum Living Standard Guarantee program. (iii) OLS estimates are reported in the table, and robust standard errors clustered at the village level are presented in parentheses. (iv) ** *p* < 0.05, * *p* < 0.1.

## Data Availability

The data presented in this study are available on request from the corresponding author. The data are not publicly available due to privacy restrictions imposed on the research team by collaborators in China.

## Supplementary Materials

The following are available online at https://www.mdpi.com/1660-4601/18/4/1457/s1, Table S1: Perceived Importance and Knowledge of Reading Practices among Caregivers.
