# Supplementary material for: Early Childhood Reading in Rural China and Obstacles to Caregiver Investment in Young Children: A Mixed-Methods Analysis
Source: Int J Environ Res Public Health. 2021 Feb 4;18(4):1457. doi: 10.3390/ijerph18041457 (PMC7913908; doi:10.3390/ijerph18041457)
Supplement: Supplementary file 1 [file ijerph-18-01457-s001.pdf]

Supplementary Material

Table S1. Perceived Importance and Knowledge of Reading Practices among Caregivers (N = 1748)

Variable	N (%)
Perceived importance of reading to child	
(1) Totally unimportant	58 (3%)
(2) Unimportant	129 (7%)
(3) Relatively unimportant	165 (9%)
(4) Neutral	479 (27%)
(5) Relatively important	465 (27%)
(6) Important	370 (21%)
(7) Extremely important	82 (5%)
Do you know how to read to your child?	
(8) Not at all	215 (12%)
(9) Not confident	231 (13%)
(10) A little unconfident	155 (9%)
(11) Neutral	335 (19%)
(12) A little confident	323 (18%)
(13) Confident	209 (12%)
(14) Absolutely confident	280 (16%)

Data Source: Authors' survey Note:

(i) The sample size N = 1748 refers to 1748 caregiver-child dyads included in the study.

(ii) The answer choices followed a 7-point Likert scale, with 1 being "unimportant" or "not knowledgeable at all" and 7 being "extremely important" or "absolutely knowledgeable"
